# Delayed anxiety and depressive morbidity among dengue patients in a multi-ethnic urban setting: first report from Sri Lanka

**DOI:** 10.1186/s13033-018-0202-6

**Published:** 2018-05-02

**Authors:** Nayana Gunathilaka, Miyuru Chandradasa, Layani Champika, Shirom Siriwardana, Lakmini Wijesooriya

**Affiliations:** 1 Department of Parasitology, Faculty of Medicine, University of Kelaniya, Ragama, Sri Lanka; 2 Department of Psychiatry, Faculty of Medicine, University of Kelaniya, PO Box 6, Thalagolla Road, Ragama, 11010 Sri Lanka; 3 Colombo North Teaching Hospital, Ragama, Sri Lanka; 4 Department of Anatomy, Faculty of Medicine, University of Kelaniya, Ragama, Sri Lanka; 5 Department of Medical Microbiology, Faculty of Medicine, University of Kelaniya, Ragama, Sri Lanka

**Keywords:** Dengue, Depression, Anxiety, Psychiatry, Sri Lanka

## Abstract

**Background:**

Although the physical consequences of dengue are well documented, delayed psychological co-morbidities are not well studied to date. Therefore, the objective of the present study was to determine the prevalence of depressive, anxiety and stress symptoms among past dengue patients.

**Methods:**

A community-based, case–control study in a multi-ethnic urban setting was conducted in Sri Lanka involving adults who were diagnosed to have dengue fever by a positive dengue IgM antibody response between 6 and 24 months ago. Self-administered Depression, Anxiety and Stress Scale (DASS-21), Centre for Epidemiological Studies Depression Scale (CESD-20) and a structured clinical interview by a psychiatrist were done in the patients and in an age and gender-matched control group.

**Results:**

Fifty-three participants each in the patient (mean age 42.9 years, SD 15.5) and control (mean age 41.6 years, SD 15.3) groups were surveyed. The ages ranged from 18 to 70 years and 64.2% were females. The majority (90.6%; n = 48) of the individuals had been diagnosed with dengue fever followed by dengue haemorrhagic fever (9.4% n = 5). Dengue patients had higher DASS-21 mean depressive scores (means 11.7/9.4, SD 6.4/4.0, t = 2.2, p = .028), anxiety scores (means 10.7/7.2, SD 6.8/1.8, t = 3.6, p = .0005), stress scores (means 12.0/8.8, SD 5.3/3.5, t = 3.6, p = .0004) and CESD-20 scores (means 16.1/11.7, SD 9.4/7.3, t = 2.6, p = .008) than controls. The DSM-5 depressive disorder was clinically detected by the psychiatrist among 15.1 and 7.5% in patient and control groups (OR 2.1; CI .5–7.7; p = .22). Limitations: a limitation is the small sample size.

**Conclusion:**

Patients with past dengue had significantly higher depressive, anxiety and stress symptoms than the control group according to the DASS-21 and CESD-20 tools. To our knowledge, this is the first report on delayed psychological morbidity related to dengue. This may warrant healthcare professionals to incorporate mental counselling for dengue patients.

## Background

Dengue is a mosquito-borne viral disease endemic in tropical and subtropical countries of the world. This has arisen as a serious international public health hazard with almost half of the world’s population at risk of infection [1]. Infection with any of the four serotypes of dengue virus (DENV 1–4) can produce a wide spectrum of symptoms, ranging from asymptomatic infection to a severe life-threatening illness. Symptomatic dengue illness is typically classified into dengue fever (DF), which is a self-limiting febrile illness, or a more severe form, dengue haemorrhagic fever (DHF)/dengue shock syndrome (DSS) [2].

This disease has been recognised as a regular epidemic since 1989, indicating an exponential increase in the incidence within Sri Lanka [3]. At present, dengue has become a serious public health concern in Sri Lanka. During 2016, 54,364 suspected dengue cases have been reported to the Epidemiology Unit of the Ministry of Health, Sri Lanka from all over the island indicating the highest ever number of cases per year [4].

Annually a considerable proportion of the health budget is allocated for the management of dengue patients and controlling of dengue vectors. The highest number of cases is reported generally from the age group of 25–49 years followed by 15–24 years [5–8]. These age groups are considered as the active workforce in the country, with significant contributions to the national development. In addition, dengue is considered as a leading cause of hospitalisation and death in children and adults [9]. Importantly, better health is central to human happiness and well-being. It also makes an important contribution to economic progress, as healthy populations live longer, are more productive, and save more. Poor health status could portend great hardships in daily activities, including monetary expenditures, loss of labour, loss of days and sometimes death.

The physical condition of individuals greatly affects their psychological health [10]. According to previously published studies, it is well known that dengue infection could affect the central nervous system of the body [11, 12]. This means that it could potentially have psychiatric manifestations during the course of the disease and they are at a greater risk of experiencing a wide range of psychological conditions such as depression, anxiety, and distress at twice the rate of the general population [10, 13].

There have been case reports of psychiatric manifestations in patients with dengue fever during the acute stage [14]. A study done in Pakistan has reported a significant prevalence of anxiety and depressive symptoms in patients diagnosed with dengue fever [15]. Further studies indicate that patients suffering from dengue fever had developed conditions such as phobic disorders and post-traumatic stress disorder [10, 16]. However, many of these studies had been done in the acute clinical setting where the patients were admitted for treatment. It is prudent to speculate that the stressors of having a physical illness and admitting to a hospital may have contributed to these acute psychological manifestations. Further to this, the physical impacts of dengue infections are adequately documented and researched; the long-term psychological impact of dengue infection remains largely unexplored.

Sri Lanka is a developing South-Asian nation with limited mental health resources. The psychiatrist to population ratio has been low compared to the west. However, with the end of the 30-year-old civil war in the North, the number of psychiatrists has risen [17]. The mental health literacy among the general population appears to be low and many major psychiatric disorders such as schizophrenia present to the services after a substantially longer duration of untreated psychosis [18].

There is no published study on psychological effects of dengue fever in Sri Lankan people. Further, there is no published information on delayed depressive and anxiety morbidity of dengue fever in the world other than few case reports mainly in acute and intermediate stages of the illness. Certain infectious aetiologies such as dengue can give rise to delayed neuropsychiatric morbidity [19]. Therefore, it is rational to assume that dengue virus infection could cause acute and delayed psychological manifestations in the patients affected. There is reasonable evidence of acute psychological disturbances in such patients and it is possible that these manifestations progress after the physical symptoms have recovered. This is important as in many physical diseases; the presence of comorbid psychopathology has proven to hinder the outcome and the functional recovery of the patient [20].

Therefore, the objective of the present study was to determine the delayed psychological morbidity associated with dengue infection. Hence, a study of this nature would be essential to explore the multidisciplinary effects of dengue infections and adopt suitable patient care and management procedures.

## Methods

### Study setting

Gampaha District is in the Western Province of Sri Lanka comprising middle sized provincial towns, semi-urban and rural areas. The district extends over 1387 km^2^ and is the second most populous district in the country. In 2012, the population of Gampaha District was approximately 2.2 million and had a population density of 1800 inhabitants/km^2^. The study was conducted in the Ragama Medical Officer of Health (MOH) area, which is one of the highest dengue prevalent areas in the District of Gampaha [21].

### Selection of study participants

Patient records of dengue-infected individuals from 2015 to 2016 were obtained from Ragama MOH office. All patients residing in Ragama MOH division who have been diagnosed with dengue fever by the fulfilment of clinical criteria for probable dengue fever according to the guidelines of the World Health Organization (WHO), confirmed by a positive IgM antibody test and notified to the Ragama MOH office by a medical practitioner were considered for the study. Individuals who were diagnosed with dengue fever during previous 2 years and whom at least 6 months had passed since the diagnosis were included. The 6-month period criteria were incorporated to minimise the effect of acute and intermediate psychological impacts of being diagnosed with a life-threatening disorder, impaired physical health, and hospitalisation. An age (± 5 years) and gender-matched control group was selected from the same community at a 1:1 ratio. Those who are under 18 years of age or with a known mental illness were excluded.

### Assessment of depressive, anxiety and stress symptoms among dengue patients

The presence of depressive, anxiety and stress symptoms was assessed by the Depression Anxiety Stress Scale 21 item version (DASS-21) [22]. The Centre for Epidemiological Studies Depression Scale 20 item version (CESD-20) was used for further detection of depressive symptoms in all the participants [23]. The DASS-21 and CESD-20 self-rated tools have been validated for the Sri Lankan population. There were no previous publications on delayed depressive and anxiety morbidity in dengue fever. Therefore, the researchers did not have knowledge of the suitability of a specific assessment tool for this unique patient group and two rather than one culturally validated freely available tools were used. The assessment tools were printed in English and local languages (Sinhala and Tamil) and were used through direct interviews following informed written consent. Subsequently, all participants were assessed clinically with the structured clinical interview for the Diagnostic and Statistical Manual of Mental disorders (DSM) by a psychiatrist. For all participants, three forms of assessments via DASS-21, CESD-20 and clinical psychiatric evaluation was done as this was the first time this unique population was studied. Details related to medical diagnosis, investigation findings and management were acquired from patient records.

### Data processing and analysis

The collected data was entered into the EPIDATA software package. Access to the data was restricted only to the investigators. The accuracy of data was routinely checked by cross-tabulations and logical checks. Discrepant data were checked against original data forms and, if any mistakes were found, were promptly corrected. The tabulated data were analysed using the Social Sciences (SPSS) software package. Descriptive statistics and frequencies were calculated for each assessed parameter. The individual scores for each sub-scale and full-scale were calculated. The mean for each sub-component was compared between groups using the independent t test method.

### Ethical aspect

Ethics clearance for the study was obtained from the Ethical Review Committee (ERC) of the Faculty of Medicine, University of Kelaniya, Sri Lanka. Informed written consent from all participants was taken.

## Results

### Socio-demographic profile of the study and control population

The present study included 53 participants from the ages of 18–70 years, with a mean age of 42.9 years (SD 15.5) in the test group. The age and gender-matched control group of 53 participants had a mean age of 41.6 years (SD 15.3). In both groups 64.2% of the recruited were females. The detailed demographic information among both study and control groups are given in Table 1.

**Table 1:** Socio-demographic data of the study and control group participants

Variable	Category	Study group (n = 53)	Control group (n = 53)
Age	18–35 years	32% (17)	37% (20)
	35–65 years	57% (30)	54% (28)
	> 65 years	11% (06)	9% (5)
Gender	Female	64% (34)	64% (34)
	Male	36% (19)	36% (19)
Ethnicity	Sinhalese	77% (41)	70% (37)
	Tamil	8% (4)	9% (5)
	Muslim	9% (5)	13% (7)
	Burgher	6% (3)	8% (4)
Medical disorder	Diabetes mellitus	11% (6)	8% (4)
	Hypertension	6% (3)	6% (3)
	Dyslipidaemia	4% (2)	6% (3)
	Hypothyroidism	4% (2)	4% (2)
	Ischaemic heart disease	6% (3)	4% (2)

Among the participants, 57% of the study group and 54% of the control group were between 35 and 65 years. A majority of the participants were of Sinhalese ethnicity, while others were Tamil, Muslim, and Burgher, respectively. Out of the study group, about 23% had been diagnosed with a medical disorder that required long-term follow-up, while it was 21% in the control group.

### Dengue infection and associated symptoms

Among the study group, 9.4% (n = 05) had been infected dengue for the second time in their life. According to the medical diagnosis, the majority (91%) of them (n = 48) were diagnosed with dengue fever followed by dengue haemorrhagic fever (Table 2). There were no individuals diagnosed with dengue shock syndrome among the study population. The mean duration from diagnosis was 14.9 months (SD 5.3). Certain symptoms that would indicate possible central nervous system involvement was inquired from the participants. Loss of consciousness and altered level of consciousness were seen among 8 and 2% respectively during the time they suffered the dengue infection (Table 2). Complications in the form of encephalopathy, liver failure, and respiratory failure were recorded in medical reports of some of the study group participants (Table 2).

**Table 2:** Data related to medical diagnosis and investigations of study group participants

Variable	Category	Percentage (n = 53)
Diagnosis	Dengue fever	91% (48)
	Dengue haemorrhagic fever	9% (5)
	Dengue shock syndrome	–
Duration from diagnosis	Less than 12 months	36% (19)
	More than 12 months	64% (34)
Significant clinical features	High fever (> 40 °C)	42% (22)
	Altered level of consciousness	2% (1)
	Loss of consciousness	8% (4)
	Seizures	–
Haematological parameters	Lowest platelet count mean value/µl (SD)	61 (28)
	Lowest white cell count mean/µl (SD)	4862 (1593)
Medical complications	Encephalopathy	13% (7)
	Respiratory failure	2% (1)
	Liver failure	2% (1)
	Cardiac failure	–
	Multi-organ failure	–

### Psychiatric symptoms

In regards to the psychological symptoms as assessed by the structured tools, the study group had significantly higher mean values for depressive, anxiety, stress subscales of DASS-21 and CESD-20 score, compared to that of the controls. The DSM-5 depressive disorder was clinically detected by the psychiatrist among 15.1% (n = 08) and 7.5% (n = 04) in study and control groups, which the difference was not statistically significant. The mean values from the assessment tools are shown in Table 3.

**Table 3:** Psychological symptom scores of the study and control group participants

Assessment	Subscale	Study group mean (SD)	Control group mean (SD)	Significance
DASS-21	Depressive	11.7 (6.4)	09.4 (4.0)	t = 2.2, p = .0287
	Anxiety	10.7 (6.8)	07.2 (1.8)	t = 3.6, p = .0005
	Stress	12.0 (5.3)	08.8 (3.5)	t = 3.6, p = .0004
CESD-20	Total	16.1 (9.4)	11.7 (7.3)	t = 2.6, p = .0083

Assessment	Disorder	Study group percentage	Control group percentage	Significance
Structured clinical interview	DSM depressive disorder	15.1%	7.5%	OR = 2.1, p = .22

Further, statistical tests were carried out in order to identify the correlations between age, duration of diagnosis, lowest platelet count and white cell count detected during the infection with psychological symptom scores. The lowest white cell count was negatively correlated with DASS depressive, stress, total and CESD-20 scores, though not significant. The correlations of the clinical parameters with the psychological scores among the study population are illustrated in Table 4. There was a significant association between the depressive symptom scores by the DASS-21 and CESD-20 with the lowest platelet count recorded in the participants during the acute infection.

**Table 4:** Correlation of clinical parameters with psychological symptom scores of the study group participants

Spearman’s correlation coefficient	DASS-21 Depressive score	DASS-21 Anxiety score	DASS-21 Stress score	DASS-21 Total score	CESD-20 Total score
Age in years	.186	.035	.092	.142	.138
p value	.181	.802	.511	.310	.323
Duration from diagnosis	.020	.023	.112	.058	-.089
p value	.888	.871	.426	.680	.527
Lowest platelet count	.350	.142	.140	.268	.286
p value	.010	.309	.316	.052	.038
Lowest white cell count	− .104	.106	− .077	− .032	− .129
p value	.460	.448	.584	.819	.357

## Discussion

People living with mental illness are at greater risk of experiencing a wide range of physical health problems. The reverse relationship is also true for people living with chronic physical health conditions where they experience depression and anxiety at twice the rate of the general population [13]. Psychiatric complications ascend during the course of a general medical condition provide an interface and opportunity to study the organic basis of psychiatric disorders [24].

Dengue is a vector-borne infection causing significant morbidity and mortality in Sri Lanka. Physical impacts of dengue are adequately documented. However, there are no published studies on delayed depressive and anxiety morbidity associated with dengue. A study conducted by Jhanjee et al. [13] has reported that the most of the patients suffering from dengue fever demonstrated significant psychiatric morbidities. The psychiatric symptoms encountered in the acute phase of dengue fever were fear of death followed by anxiety and associated symptoms.

The current study compared the delayed psychological morbidity in the form of depressive, anxiety, stress symptoms assessed by structured tools and the presence of depressive disorder clinically by a psychiatrist in adults living in the community who had a diagnosis of dengue fever between 6 and 24 months before assessment in comparison to that of age and gender-matched controls. The findings revealed that participants who had dengue fever previously had significantly higher depressive, anxiety, stress symptoms as measured by DASS-21 and depressive symptoms as measured by CESD-20, compared to that of the controls. A higher percentage of depressive disorder diagnosed by the psychiatrist via clinical assessment was detected among the persons who had dengue fever, but the difference was not significant as compared to the control group.

A study conducted in 2012, in Pakistan, found that more than 60% of the patients met criteria for depression and anxiety during their hospitalisation. As in the current study, the psychological symptoms were in higher proportionate to certain clinical parameters such as low platelet count [15]. In our study, the presence of depressive morbidity according to the DASS-21 and CESD-20 was significantly associated with the lowest platelet count recorded during the infection. In another study from Pakistan, which was conducted in 2014, the researchers assessed psychological distress, resilience, and well-being among 100 survivors of dengue fever. They found that psychological distress was negatively associated with the well-being of the participants. There was no evaluation of depressive, anxiety symptoms and no comparisons with controls were done [25].

There are case-reports of mania and psychosis associated with dengue infection [26, 27]. Even though all participants were assessed clinically by a psychiatrist in the current study, mania and psychosis were not detected, including psychotic features associated with depression. It is possible that these psychiatric manifestations are rare associations of dengue. Some have argued that psychiatric manifestations are rarely reported in association with dengue due to lack of awareness among clinicians or due to no clinical association with dengue [28].

With respect to the possible causation of depressive and anxiety morbidity in dengue, it is highly likely due to stresses of hospitalisation and physical morbidity in the acute and intermediate stages of the infection. However, these elements are unlikely to play a role in causing psychological morbidity in the long term as assessed in this study. A prudent assumption would be that either organic brain changes due to the infection or psycho-social factors related to dengue are responsible. It is known that the Sri Lankan population had experienced severe dengue epidemics in the past and they were associated with high mortality and fear of dengue in the community [29]. With respect to possible organic causation, altered consciousness, generalised convulsions, coma with generalised irregular slow waves in encephalograms in serology positive dengue patients have been reported from Sri Lanka. These clinical and investigative features suggest cerebral involvement and potential for psychiatric manifestations of dengue in association with clinical and subclinical encephalitis [30].

### Strengths and limitations

A limitation of the current study is the small sample size. Patients who had dengue are not required to attend any medical follow-up; they are unaware of possible psychiatric manifestations and live in the community physically unaffected at present. Therefore, it is difficult to recruit such patients for a study on psychological morbidity and these factors probably justify the sample size. Further to this, the groups were not controlled for the educational level of the participants. This might be a limitation when assessing for depression and anxiety as the higher educational level has shown to be a protective factor for developing these conditions throughout life [31].

In supposition, persons with past dengue infection had significantly higher depressive and anxiety morbidity according to the DASS-21 and CESD-20 tools compared to age and gender-matched controls in a multi-ethnic urban population in Sri Lanka. This is the first study to report on the delayed depressive and anxiety morbidity associated with dengue infection. These findings indicate the importance of awareness among health-care professionals and community about the possibility of psychological complications after dengue fever. Further longitudinal research may be required to explore the progression of psychological morbidity and aetiological factors.

## Conclusion

This study revealed that patients with past dengue infection had significantly higher depressive, anxiety, and stress symptoms according to the sub-scales of the DASS-21 and higher depressive symptoms according to the total score of the CESD-20 compared to the control group. Clinically diagnosed depressive disorder by the psychiatrist according to DSM was higher among dengue patients, though not significant compared to the controls. Observations noted from the present study are the first time for delayed psychological morbidity in dengue. This may warrant the healthcare professionals to incorporate mental assessment and counselling for dengue patients.
